# Matrons' Corner

**Published:** 1887-11-12

**Authors:** 


### THE MATRONS' CORNER.

[Replies, endorsed "Matron," should be addressed to the Editor.]

### RULES FOR PROBATIONERS BEGINNING WARD WORK.

I beg to say, in reference to my letter of last week regarding "Rules for Probationers Beginning Ward Work," that I have since had sent to me, through the kindness of the matron and of a former probationer at Brownlow Hill, complete copies of the rules to which I referred, and which I find are even more admirably clear, precise, and comprehensive than I had remembered them to be. I shall have much pleasure in sending a copy of these rules to any matron if she will, in writing to me, kindly enclose a stamped and addressed envelope (folio size).—I am, truly yours,

Jane Wilson, Hon. Sec.,
Workhouse Infirmary Nursing Association.

45, Colville Gardens, W.

[Would Miss Wilson kindly send us a copy in duplicate?—Ed. *T. H.*]

